# Draft genome sequences of two bovine-associated *Lachnospiraceae*: *Pseudobutyrivibrio xylanivorans* DSM 14809^T^ and [*Clostridium*] *fimetarium* DSM 9179^T^

**DOI:** 10.1128/mra.00875-26

**Published:** 2026-08-31

**Authors:** Samantha Marroquin, Tanmay Rohatgi, Stefan Spring, Markus Göker, Chris Daum, Marcel Huntemann, Rekha Seshadri, Matthew A. Escobar

**Affiliations:** 1 Department of Biological Sciences, California State University San Marcos 14673, San Marcos, California, USA; 2 Leibniz Institute DSMZ, German Collection of Microorganisms and Cell Cultures 28351, Braunschweig, Germany; 3 Department of Energy Joint Genome Institute, Lawrence Berkeley National Laboratory 118576, Berkeley, California, USA; USDA-ARS San Joaquin Valley Agricultural Sciences Center, Parlier, California, USA

**Keywords:** genome, bovine, cellulose, xylan

## Abstract

Here, we report draft genome sequences of two bacterial type strains isolated from cows, *Pseudobutyrivibrio xylanivorans* DSM 14809^T^ and [*Clostridium*] *fimetarium* DSM 9179^T^. The *P. xylanivorans* DSM 14809^T^ genome is 3.4 Mbp with 3,147 predicted genes, while the *C. fimetarium* DSM 9179^T^ genome is 4.6 Mbp with 4,215 predicted genes.

## ANNOUNCEMENT

Bacteria capable of fermenting plant-derived polysaccharides are central to the nutrition of ruminant animals (1, 2). The saccharolytic type strains *Pseudobutyrivibrio xylanivorans* DSM 14809^T^ (Mz 5^T^) and [*Clostridium*] *fimetarium* DSM 9179^T^ (Z-2189^T^) were originally isolated from cow rumen fluid in Domžale, Slovenia, and cow manure in Russia, respectively (3–5). Both species are flagellated, obligately anaerobic bacilli in the family *Lachnospiraceae*, but *P. xylanivorans* DSM 14809^T^ is gram-negative, while *C. fimetarium* DSM 9179^T^ is gram-positive (4, 5). The draft genome sequences of these strains will expand our understanding of ruminant microbiology and physiology.

Cultures were grown anaerobically for 1 day at Leibniz Institute DSMZ using Medium 330 at 40°C (*P. xylanivorans*) or Medium 500 at 20°C (*C. fimetarium*) (6). DNA was isolated from both cultures using a MasterPure Gram-Positive DNA Purification Kit (Epicentre) according to the manufacturer’s protocol, except for the use of 2 µL of proteinase K (protocol step 5) and an overnight incubation on ice with agitation (protocol step 9). Genome sequencing was performed by the Joint Genome Institute as part of the KMG-I and KMG-II projects (7, 8). DNA was sheared to 270–300 bp using a Covaris LE220 sonicator and size-selected using SPRI beads (Beckman Coulter). Fragments were end-repaired, A-tailed, and ligated with Illumina-compatible adapters using a KAPA-Illumina library kit (KAPA Biosystems). The library was quantified using KAPA Biosystems’ NGS library qPCR kit (Roche) with a Roche LightCycler 480. The flow cell was prepared using an Illumina TruSeq paired-end cluster kit with a cBot instrument (Illumina). Sequencing was performed on an Illumina HiSeq2000 (*P. xylanivorans*) or HiSeq2500 (*C. fimetarium*) using HiSeq TruSeq SBS sequencing kits (Illumina) with a 2 × 150-bp indexed run. For *P. xylanivorans*, 10,760,860 reads were generated (1,614 Mbp) and then filtered using DUK (v1.0) (9), exactly as previously described (10). Filtered reads were assembled using Velvet (v1.2.07), wgsim (v1.0), and Allpaths-LG (v.r46652) (11–13), and the genome was then annotated using Prodigal (v2.5) and GenePRIMP (v1.0) (14, 15), with all software settings as previously described (16). For *C. fimetarium*, 7,942,060 reads were generated (1,191 Mbp) and then filtered using BBDUK (v1.0) (17), exactly as previously described (18). SPAdes (v.3.6.2; -cov-cutoff auto --phred-offset 33 -t 8 -m 40 --careful -k 25,55,95 --12) (19) was used for genome assembly, and contigs <1,000 bp were discarded. Annotation was performed using the IMG Genome Annotation Pipeline (v.4.11.2) with default parameters (20, 21). The features of both genomes are summarized in Table 1.

**TABLE 1:** Genomic features of *P. xylanivorans* DSM 14809^T^ and *C. fimetarium* DSM 9179^T^

Genome feature	*P. xylanivorans* DSM 14809^T^	*C. fimetarium* DSM 9179^T^
Draft genome size (bp)	3,420,924	4,568,183
Number of scaffolds	57	48
Scaffold N_50_ (bp)	156,596	228,153
Average genome coverage	438×	261×
CheckM2 genome completeness (22)	99.93%	98.97%
CheckM2 genome contamination (22)	0.24%	2.29%
GC content (%)	38.72	33.59
Predicted genes	3,147	4,215
Predicted rRNAs	6	9
Predicted tRNAs	55	49
Genes with GH5 domains (putative cellulase)	4	2
Genes with GH8 domains (putative cellulase)	1	0
Genes with GH9 domains (putative cellulase)	3	0
Genes with GH44 domains (putative cellulase)	0	0
Genes with GH48 domains (putative cellulase)	0	0
Genes with GH10 domains (putative xylanase)	2	0
Genes with GH11 domains (putative xylanase)	1	0
Joint Genome Institute IMG/M taxon ID	2585428087	2675903166
NCBI WGS accession number	GCA_900141825.1	GCA_900111235.1
NCBI BioProject accession number	PRJNA245713	PRJNA329848
NCBI Sequence Read Archive accession number	SRR4096530	SRR4233938
NCBI BioSample number	SAMN02745725	SAMN05421659

While *P. xylanivorans* DSM 14809^T^ can hydrolyze plant-derived polysaccharides such as xylan and cellulose, *C. fimetarium* DSM 9179^T^ cannot (4, 5). Accordingly, *P. xylanivorans* DSM 14809^T^ has eight putative cellulase genes (with GH5/8/9/44/48 domains) and three putative xylanase genes (with GH10/11 domains), while *C. fimetarium* DSM 9179^T^ has two putative cellulase genes and no putative xylanase genes. Notably, two other genome sequences currently classified as “*P. xylanivorans*,” strains DSM 10317 and MA3014 (23), do not meet the average nucleotide identity threshold for species delineation (24) compared with the type strain *P. xylanivorans* DSM 14809^T^, and may require reclassification.

## Data Availability

This whole-genome shotgun project has been deposited in GenBank under the accession numbers GCA_900141825.1 (*P. xylanivorans* DSM 14809^T^) and GCA_900111235.1 (*C. fimetarium* DSM 9179^T^). Additional publicly available genome resource links are included in Table 1.
